# Effect of Intrinsic and Extrinsic Factors on Global and Regional Cortical Thickness

**DOI:** 10.1371/journal.pone.0096429

**Published:** 2014-05-02

**Authors:** Koushik A. Govindarajan, Leorah Freeman, Chunyan Cai, Mohammad H. Rahbar, Ponnada A. Narayana

**Affiliations:** 1 Department of Diagnostic and Interventional Imaging, The University of Texas – Health Sciences Center, Houston, Texas, United States of America; 2 Division of Clinical and Translational Sciences, Department of Internal Medicine, University of Texas Medical School at Houston, The University of Texas – Health Sciences Center, Houston, Texas, United States of America; 3 Division of Epidemiology, Human Genetics and Environmental Sciences, School of Public Health, The University of Texas – Health Sciences Center, Houston, Texas, United States of America; University of California, San Francisco, United States of America

## Abstract

Global and regional cortical thicknesses based on T1-weighted magnetic resonance images acquired at 1.5 T and 3 T were measured on a relatively large cohort of 295 subjects using FreeSurfer software. Multivariate regression analysis was performed using Pillai's trace test to determine significant differences in cortical thicknesses measured at these two field strengths. Our results indicate that global cortical thickness is not affected by the field strength or gender. In contrast, the regional cortical thickness was observed to be field dependent. Specifically, the cortical thickness in regions such as parahippocampal, superior temporal, precentral and posterior cingulate is thicker at 3 T than at 1.5 T. In contrast regions such as cuneus and pericalcarine showed higher cortical thickness at 1.5 T than at 3 T. These differences appear to be age-dependent. The differences in regional cortical thickness between field strengths were similar in both genders. Further, male vs. female differences in regional cortical thickness were observed only at 1.5 T and not at 3 T. Our results indicate that magnetic field strength has a significant effect on the estimation of regional, but not global, cortical thickness. In addition, the pulse sequence, scanner type, and spatial resolution do not appear to have significant effect on the measured cortical thickness.

## Introduction

Global and regional cortical thicknesses provide valuable insight into normal brain development and the effect of various neurological and neuropsychiatric disorders. Magnetic resonance imaging (MRI) is most commonly used for measuring cortical thickness in vivo. Cortical thickness depends on various factors such as age and gender and has been extensively studied [1]–[10]. Cortical thickness is an intrinsic biological parameter and should be independent of external factors such as the MRI scanner type, imaging sequence, spatial resolution and/or field strength. Previously published studies evaluated the effect of such external factors on the measured cortical thickness. For example, study by Han et al [11], based on a small population of 15 subjects, reported that the mean or global cortical thickness was up to 0.17 mm higher at 3 T compared to 1.5 T. Though the reasons for this difference are not entirely clear, it is possible that the superior contrast to noise ratio (CNR) at 3 T over 1.5 T results in more accurate measurements of cortical thickness that could explain the field-dependent cortical thickness. Also, Wonderlick et al [12], again based on a small sample of 11 subjects, evaluated the effect of MR pulse sequence, resolution and parallel imaging techniques on the estimation of cortical thickness and concluded that cortical thickness and volumetric measurements were reliably reproduced across differences in acquisition.

Majority of the published cortical thickness measurements were performed at 1.5 T. With ever increasing use of 3 T scanners, it is important to investigate if the global and regional cortical thickness measurements are dependent on the field strength. It is also important to investigate if the field strength dependence of cortical thickness is modulated by age and gender. Also, much of the published literature on evaluating the reliability of cortical thickness measurements is based on small sample sizes and fails to mimic the heterogeneity of multi-center clinical trials. In an earlier study that mainly focused on comparing cortical thickness between relapsing-remitting multiple sclerosis (MS) and normal controls, we investigated cortical thickness, its age-dependence and the effect of gender and field strength in a sample of 125 normal controls [13]. In that study, the controls were selected to age- and gender match the MS cohort. That study lacked adequate sample size to properly evaluate the effect of gender at each field strength and the effect of field strength separately in each gender group in the control population. In order to better evaluate the effect of gender and field strength on the measured cortical thickness in the current study we measured both global and regional cortical thicknesses measured at 3 T and 1.5 T on a relatively large cohort of 295 normal controls.

## Materials and Methods

This is a retrospective analysis of the anonymized MRI data on normal volunteers accessed from various publicly available databases, except for the 56 datasets acquired at our center. The MRI protocol on the 56 subjects was approved by the Committee for the Protection of Human Subjects (CPHS) at UTHealth at Houston. Written informed consent was obtained from each one of the 56 subjects. The retrospective data analysis received IRB exemption from the CPHS at UTHealth.

A total of 316 MRI datasets were selected from multiple sources including various publicly available databases. This sample consisted of 56 datasets from the 3 T Philips scanner at our facility (voxel size of 0.94 mm×0.94 mm×1 mm), 62 datasets from the OASIS public database (http://www.oasis-brains.org, voxel size of 1 mm×1 mm×1 mm) [14], 5 datasets from the IBSR database (http://www.cma.mgh.harvard.edu/ibsr/data.html, voxel size of 1 mm×1 mm×1.5 mm), 2 datasets from the Kirby research center (http://www.nitrc.org/projects/multimodal, voxel size of 0.9 mm×0.9 mm×1.5 mm) [15], 101 datasets from the ICBM (http://ida.loni.ucla.edu, voxel size of 1 mm×1 mm×1 mm), 7 datasets from the BIRN (http://www.birncommunity.org/resources/data, voxel size of 1 mm×1 mm×1 mm) and 83 datasets from the Imperial College of London (http://biomedic.doc.ic.ac.uk/brain-development/index.php; voxel size of 0.94 mm×0.94 mm×1.2 mm) databases. The datasets were carefully selected to maintain a balance between the genders (***χ*** ^2^ = 0.16) and also between field strengths while at the same time, maintaining a uniform distribution in the specified age range. All the images were reviewed by an experienced neurologist (LF with 8 years of experience) for any incidental pathology. All the T1-weighted MRI scans were resampled to an isotropic voxel of 1 mm. All the images were checked for artifacts and poor signal-to-noise ratio using an in-house developed automatic quality assurance software [13].

Images were processed using the FreeSurfer pipeline (FreeSurfer v5.1.0) on a Linux environment using a 64-bit Redhat Enterprise operating system. The methodology of the FreeSurfer pipeline has been extensively documented in the literature [16], [17] and therefore only a brief overview is provided here. The FreeSurfer pipeline essentially consists of two processing streams, a volumetric stream and a surface-based stream. After registering to the template and normalizing the intensity, the images are skull-stripped based on a combination of watershed algorithm and deformable template model [18]. The output brain mask is labeled using a probabilistic atlas. Following that, in the surface based stream, white matter (WM) segmentation is performed and then the gray matter (GM) and WM boundary and the pial surface [16], [17] are identified using a tessellation technique. Neighborhood intensity information is used to identify likely white matter voxels. White matter and pial surfaces are constructed after refining the initial surfaces generated for each hemisphere. The cortical thickness is defined as the average of the distance between the surface and the GM-WM boundary and the distance between the GM-WM boundary and the surface [19]. Spatial location of different regions is used to assign a neuroanatomical label to each region. Based on a priori knowledge of conventional neuroanatomy and combining it with geometric information based on the cortical model, the entire cortical gyri and sulci are labeled. This study used the Desikan-Killiany atlas [20] for the neuroanatomical labels. The computation time for the FreeSurfer pipeline for each subject was about 15–25 hours.

In order to evaluate the group differences in cortical thickness, separate multiple comparison Monte Carlo simulations for the 2 field strengths were performed with 5000 iterations using FreeSurfer's group analysis module. Statistical inferences were based on p = 0.001 with false discovery rate (FDR) correction.

### Statistical methods

Multivariate regression [21], [22] was performed to assess the relationship between cortical thickness and field strength. Specifically, we considered regional cortical thickness as a vector of dependent variable and treated the field strength as an independent variable. Statistical significance of field strength on the cortical thickness was evaluated using Pillai's trace test [23], [24]. Since age, gender, scanner type, image resolution, and pulse sequence are considered as potential confounding variables or effect modifiers on evaluating the relationship between field strength and cortical thickness, we added gender, age, gender*field, age*field, scanner type, image resolution and pulse sequence into our regression models as covariates. The age distribution of the subjects in this study is not uniform. Therefore, we divided the subjects into three age groups with comparable sample size. The age groups are 20–40 years (agecat1), 41–55 years (agecat2), and 56–80 years (agecat3). Using agecat1 (20–40 yrs) as the referent category, we maintained dummy variables agecat2 and agecat3 in the models to represent the age. We used fieldtype to denote the type of field strength, where fieldtype  = 0 and 1 indicate 1.5 T and 3 T field strength, respectively. For pulse sequence, we considered MPIRI and MPRAGE as one category since both are based on inversion recovery, and considered FFE and GRE as another category. For image resolution, we considered 0.94*0.94*1 and 1*1*1 as one category, and considered 0.94*0.94*1.2 and 0.94*0.94*1.5 as another category for reducing the number of covariates. The vectors of coefficients from multivariate regression model are estimated. Finally, the adjusted means and mean differences between 3 T and 1.5 T regional cortical thicknesses along with 95% confidence intervals were calculated. All the above analyses were performed using SAS 9.3 (http://www.sas.com; Cary, NC)

## Results

### Quality control

**Figure 1:**
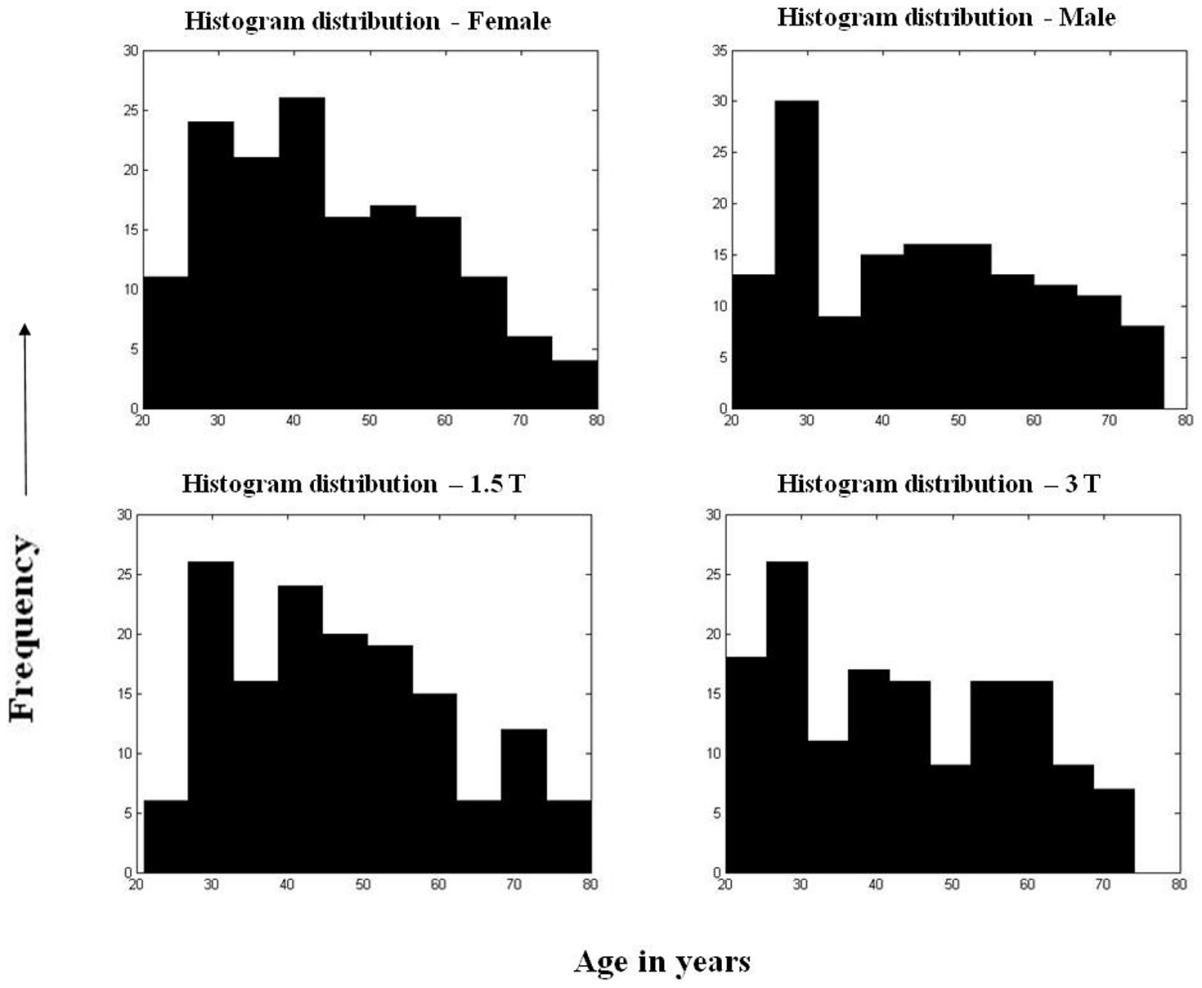
Histogram distribution for the samples – (a) Female, (b) Male, (c) 1.5 T and (d) 3 T.

Twenty one scans were excluded from the study for reasons that included the presence of focal or diffuse lesions (n = 6, 2 deep grey matter lesions, 2 focal frontal lesions and 2 extensive vascular leukopathy), poor image quality (n = 5), and segmentation failure (n = 10). No manual edits were performed on the remaining scans. The final cohort consisted of 295 datasets in the age range of 20–80 years. The demographic information on the study population is summarized in Table 1. The histogram distribution of the gender and field strength is shown in Fig. 1. Table 2 summarizes the sample distribution based on image resolution, pulse sequence, and scanner manufacturer type.

**Table 1:** Demographic information for the sample of 295 controls split into both genders and field strengths.

Field strength/Gender (N; Age ± SD; Range)	Male (143; 45.7±15.6; 20–79)	Female (152; 44.8±14.3; 20–80)
1.5 T (151; 46.9±14.7; 21–80)	74; 48.9±13.6; 27–77	77; 45.1±15.7; 21–80
3 T (144; 43.2±14.9; 20–79)	69; 41.1±16.6; 20–79	75; 45.2±12.8; 20–71

**Table 2:** Sample distribution based on image resolution, pulse sequence and scanner manufacturer type.

Parameter	Specification	Field Strength		Total
		1.5 T	3 T	
Resolution	0.94×0.94×1	0	56	56
(mm × mm × mm)	0.94×0.94×1.2	8	74	82
	0.94×0.94×1.5	1	2	3
	1×1×1	141	13	154
Pulse sequence	FFE	18	133	151
	GRE	8	6	14
	MPIRI	9	0	9
	MPRAGE	115	6	121
Scanner type *	GE	9	6	15
	Philips	18	134	152
	Siemens	121	5	126

*Statistical analysis based on scanner type excluded 2 datasets from the sample population.

### Global cortical thickness

#### Field strength and gender dependence

The global cortical thickness averaged over the 295 subjects was not statistically different at the two field strengths (2.37±0.13 mm at 3T vs. 2.35±0.15 mm at 1.5 T; p = 0.95). The global cortical thickness also did not differ across the gender: males (2.35±0.12 mm) vs. females (2.36±0.12 mm) (p = 0.98). The effect of gender on global cortical thickness at each of these two field strengths was also evaluated and those values were also not statistically significant: males (2.35±0.13 mm) vs. females (2.35±0.12 mm) at 1.5 T (p = 0.98) and males (2.34±0.11 mm) vs. females (2.37±0.14 mm) at 3 T (p = 0.95).

### Regional cortical thickness

The regional cortical thickness was measured in 68 regions (34 regions in each hemisphere). These structures included banks of the superior temporal sulcus, caudal anterior cingulate, caudal middle frontal, cuneus, entorhinal, fusiform, inferior parietal, inferior temporal, isthmus cingulate, lateral orbitofrontal, lateral occipital, lingual, medial orbitofrontal, middle temporal, parahippocampal, paracentral, pars opercularis, pars orbitalis, pars triangularis, pericalcarine, postcentral, posterior cingulate, precuneus, precentral, rostral anterior cingulate, rostral middle frontal, superior frontal, superior parietal, superior temporal, supramarginal, frontal pole, temporal pole, transverse temporal and insula. The cortical thickness over the entire cohort varied from 1.6 mm (pericalcarine) to 3.6 mm (entorhinal and temporal pole). The mean cortical thickness values of the 34 segmented regions in the two hemispheres are listed in Table 3. There were no significant differences between left and right hemisphere at p = 0.05.

**Table 3:** Mean Regional Cortical thickness for both hemispheres in 295 controls.

Region	Left Hemisphere (Mean ± SD)	Right Hemisphere (Mean ± SD)
Banks of the STS+	2.53±0.20	2.55±0.18
Caudal anterior cingulate	2.60±0.25	2.61±0.26
Caudal middle frontal	2.53±0.17	2.53±0.17
Cuneus	1.86±0.15	1.85±0.16
Entorhinal	3.28±0.37	3.29±0.38
Fusiform	2.61±0.16	2.59±0.16
Inferior parietal	2.48±0.15	2.50±0.15
Inferior temporal	2.75±0.17	2.76±0.17
Isthmus cingulate	2.48±0.21	2.42±0.21
Lateral occipital	2.15±0.15	2.18±0.15
Lateral orbitofrontal	2.64±0.18	2.66±0.17
Lingual	1.98±0.16	1.98±0.16
Medial orbitofrontal	2.48±0.19	2.47±0.19
Middle temporal	2.87±0.17	2.88±0.17
Parahippocampal	2.67±0.34	2.61±0.30
Paracentral	2.31±0.20	2.28±0.19
Pars opercularis	2.60±0.17	2.60±0.18
Pars orbitalis	2.73±0.23	2.70±0.26
Pars triangularis	2.51±0.19	2.54±0.19
Pericalcarine	1.63±0.18	1.60±0.18
Postcentral	2.04±0.14	2.04±0.14
Posterior cingulate	2.51±0.17	2.50±0.18
Precentral	2.47±0.19	2.45±0.19
Precuneus	2.35±0.15	2.35±0.15
Rostral anterior cingulate	2.86±0.26	2.87±0.27
Rostral middle frontal	2.38±0.15	2.39±0.15
Superior frontal	2.74±0.18	2.73±0.17
Superior parietal	2.18±0.15	2.20±0.15
Superior temporal	2.78±0.19	2.78±0.19
Supramarginal	2.55±0.16	2.58±0.15
Frontal pole	2.73±0.32	2.72±0.31
Temporal pole	3.58±0.41	3.66±0.40
Transverse temporal	2.35±0.27	2.34±0.28
Insula	2.98±0.18	2.98±0.19

There were no significant differences between the two hemispheres at p = 0.05.

+ STS  =  superior temporal sulcus.

#### Regional cortical thickness – field dependence

The regional cortical thicknesses at the two field strengths, averaged over the entire cohort, are summarized in Table 4. Using unadjusted means and mean differences, the regions that were significantly different between 1.5 T and 3 T were determined at p<0.05. As can be seen from this Table, significant differences were observed in a number of structures between 1.5 T and 3T and these differences seem to be bilateral. The regions with the most differences between field strengths were parahippocampal, fusiform, pericalcarine, rostral anterior cingulate, entorhinal, cuneus and the temporal pole.

**Table 4:** Unadjusted means and mean differences (95% confidence interval) of regional cortical thickness by field strength (1.5 T vs. 3 T) for right and left hemispheres in 295 controls using multivariate regression model.

Region	Left Hemisphere			Right Hemisphere		
	1.5 T	3 T	Mean differences	1.5 T	3 T	Mean differences
Banks of the STS^+^	2.50*	2.56*	0.06 (0.01, 0.10)	2.50*	2.61*	0.11 (0.07, 0.15)
Caudal anterior cingulate	2.56*	2.64*	0.08 (0.02, 0.14)	2.60	2.62	0.01 (−0.05, 0.07)
Caudal middle frontal	2.54	2.53	−0.01 (−0.05, 0.03)	2.52	2.54	0.02 (−0.02, 0.06)
Cuneus	1.91*	1.80*	−0.12 (−0.15, −0.08)	1.92*	1.78*	−0.14 (−0.18, −0.10)
Entorhinal	3.23*	3.32*	0.09 (0.01, 0.18)	3.19*	3.39*	0.20 (0.12, 0.29)
Fusiform	2.54*	2.68*	0.14 (0.10, 0.18)	2.52*	2.66*	0.14 (0.10, 0.18)
Inferior parietal	2.48	2.49	0.02 (−0.02, 0.05)	2.50	2.51	0.00 (−0.03, 0.04)
Inferior temporal	2.73	2.76	0.03 (−0.01, 0.07)	2.72*	2.80*	0.09 (0.05, 0.13)
Isthmus cingulate	2.42*	2.53*	0.11 (0.06, 0.15)	2.39*	2.45*	0.06 (0.01, 0.11)
Lateral occipital	2.18*	2.11*	−0.08 (−0.11, −0.04)	2.22*	2.15*	−0.07 (−0.10, −0.03)
Lateral oribitofrontal	2.57*	2.70*	0.12 (0.08, 0.17)	2.64*	2.69*	0.05 (0.01, 0.09)
Lingual	2.03*	1.94*	−0.09 (−0.13, −0.05)	2.04*	1.92*	−0.11 (−0.15, −0.08)
Medial orbitofrontal	2.49	2.46	−0.04 (−0.08, 0.01)	2.45	2.49	0.04 (0.00, 0.09)
Middle temporal	2.83*	2.92*	0.09 (0.05, 0.13)	2.83*	2.92*	0.09 (0.05, 0.13)
parahippocampal	2.53*	2.81*	0.28 (0.20, 0.36)	2.47*	2.75*	0.28 (0.21, 0.35)
paracentral	2.31	2.31	0.00 (−0.04, 0.05)	2.27	2.28	0.01 (−0.03, 0.06)
Pars opercularis	2.59	2.62	0.03 (−0.01, 0.07)	2.59	2.62	0.03 (−0.01, 0.07)
Pars orbitalis	2.69*	2.76*	0.07 (0.02, 0.12)	2.68	2.72	0.04 (−0.02, 0.10)
Pars triangularis	2.49*	2.53*	0.04 (0.00, 0.09)	2.51*	2.56*	0.05 (0.00, 0.09)
Pericalcarine	1.72*	1.55*	−0.17 (−0.21, −0.13)	1.68*	1.51*	−0.17 (−0.21, −0.13)
Postcentral	2.06*	2.02*	−0.04 (−0.07, −0.01)	2.07*	2.02*	−0.04 (−0.08, −0.01)
Posterior cingulate	2.46*	2.55*	0.09 (0.05, 0.13)	2.46*	2.54*	0.08 (0.04, 0.12)
Precentral	2.44*	2.50*	0.06 (0.01, 0.10)	2.42*	2.48*	0.06 (0.02, 0.11)
Precuneus	2.36	2.35	−0.01 (−0.05, 0.02)	2.35	2.35	0.01 (−0.03, 0.04)
Rostral anterior cingulate	2.79*	2.93*	0.14 (0.08, 0.20)	2.83*	2.91*	0.08 (0.02, 0.15)
Rostral middle frontal	2.40*	2.36*	−0.04 (−0.08, −0.01)	2.42*	2.37*	−0.04 (−0.08, −0.01)
Superior frontal	2.72	2.75	0.02 (−0.02, 0.06)	2.71	2.75	0.04 (0.00, 0.07)
Superior parietal	2.21*	2.15*	−0.05 (−0.09, −0.02)	2.23*	2.18*	−0.05 (−0.09, −0.02)
Superior temporal	2.75*	2.81*	0.06 (0.02, 0.11)	2.73*	2.83*	0.10 (0.06, 0.14)
Supramarginal	2.53*	2.57*	0.04 (0.00, 0.07)	2.58	2.58	0.01 (−0.03, 0.04)
Frontal pole	2.76	2.70	−0.06 (−0.13, 0.01)	2.76*	2.67*	−0.09 (−0.16, −0.02)
Temporal pole	3.47*	3.69*	0.21 (0.12, 0.31)	3.56*	3.75*	0.19 (0.09, 0.28)
Transverse temporal	2.37	2.32	−0.05 (−0.11, 0.01)	2.32	2.37	0.04 (−0.02, 0.11)
Insula	2.97	2.99	0.02 (−0.02, 0.06)	3.00	2.97	−0.02 (−0.06, 0.02)

The regions with the significant field strength effect (p<0.05) on cortical thickness are denoted with “*”.

Note: overall field strength effect on regional cortical thickness is significant with p-value <0.0001 using Pillai's Trace test. ^+^STS  =  superior temporal sulcus.

**Figure 2:**
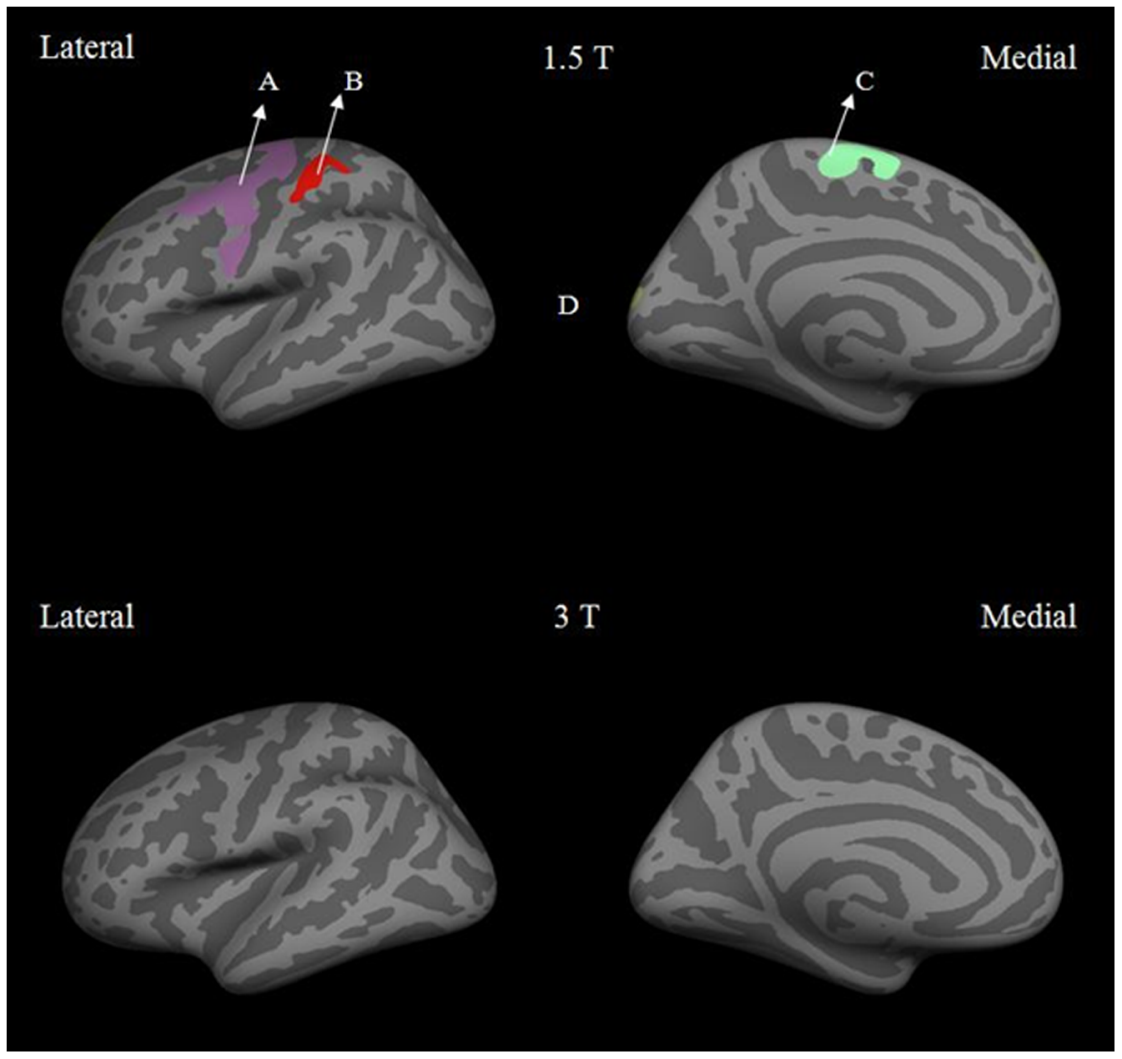
Lateral and medial views of inflated left hemisphere at 1.5 T and 3 T for male vs. female differences in cortical thickness. The labeled regions are: (A) precentral, (B) postcentral, (C) paracentral and (D) lateral occipital

**Figure 3:**
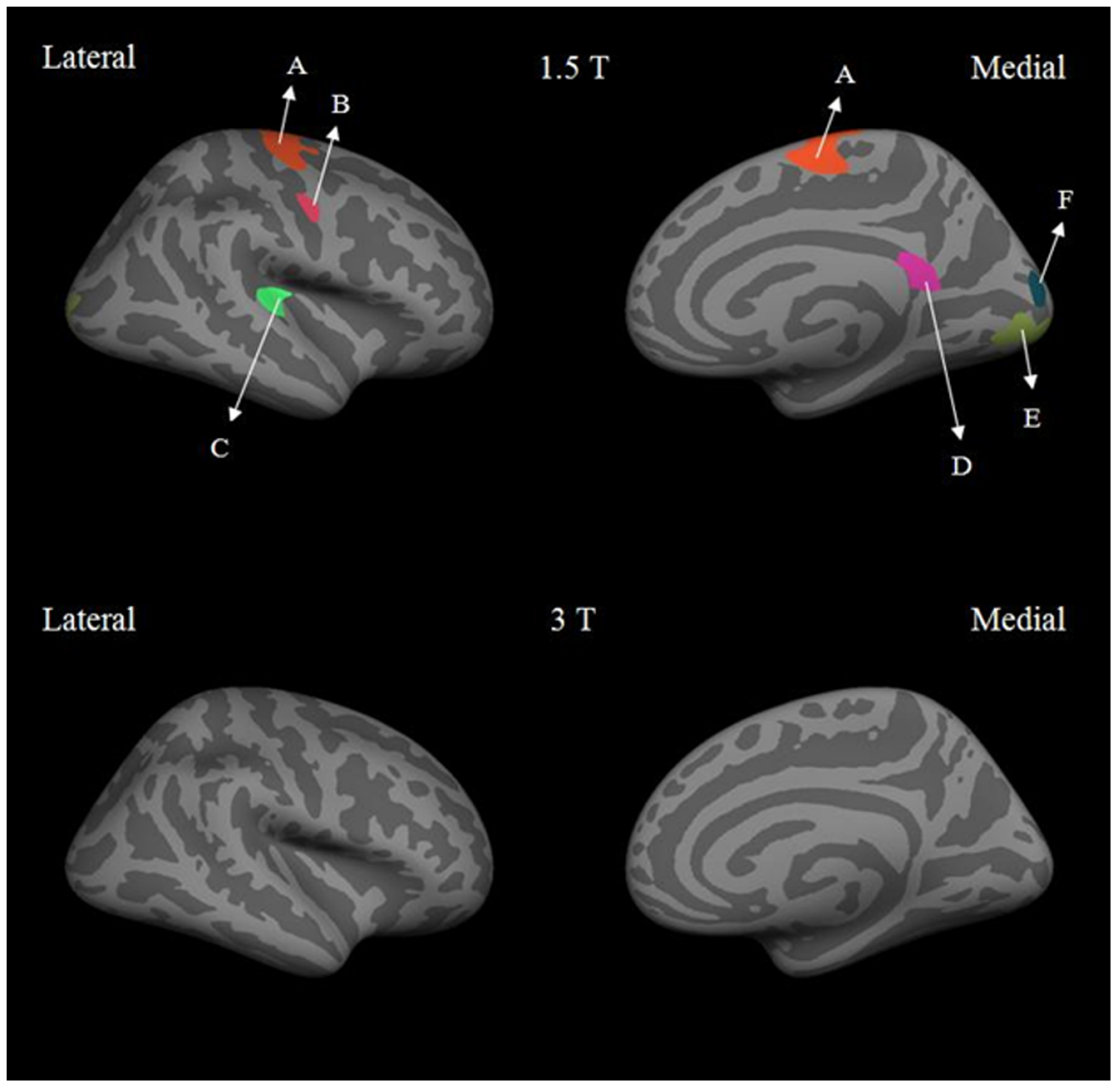
Lateral and medial views of inflated right hemisphere at 1.5 T and 3 T for male vs. female differences in cortical thickness. The labeled regions are: (A) and (B) precentral, (C) transverse temporal, (D) isthmus cingulate, (E) lingual and (F) cuneus

Based on these differences, we wanted to further determine if the dependence of cortical thickness on field strength was influenced by gender. Figures 2 and 3 show the results of male vs. female cortical thickness difference maps at 1.5 T and 3 T for the left and right hemisphere respectively. At 1.5 T, the male vs. female difference maps for the left hemisphere showed the precentral, paracentral and postcentral regions to be significantly thicker in females than in males. In the right hemisphere, the precentral, transverse temporal, isthmus cingulate, cuneus and lingual regions showed greater cortical thickness in females than in males. In contrast, the male vs. female difference maps for both hemispheres at 3 T showed no significant differences. Overall, the mean cortical thickness was found to be up to 0.4 mm thicker at 3T compared to 1.5 T in regions such as parahippocampal, superior temporal, precentral and posterior cingulate in females.

In order to further explore if these observed effects of field strength on cortical thickness were influenced by age, we divided the cohort into 3 age groups (20–40 yrs, 41–55 yrs and 51–80 yrs). Using mean differences of regional cortical thickness adjusted by age and gender, data from 1.5 T and 3 T were compared for both genders for the three age groups. In the younger group (20–40), the following regions showed significantly higher cortical thickness at 3 T than at 1.5 T in both male and female cohorts: banks of the superior temporal sulcus, fusiform, isthmus cingulate, middle temporal, parahippocampal, paracentral, posterior cingulate, precentral, superior temporal, transverse temporal, temporal pole and supramarginal. For the 41–55 age group, only the fusiform, frontal pole and parahippocampal regions showed significantly higher cortical thickness at 3 T compared to 1.5 T for both genders. In contrast, cortical thickness measured at 1.5 T was significantly higher than at 3 T for the following regions for the age groups 41–55 and 56–80: cuneus, pericalcarine, superior parietal and transverse temporal. In addition, for the 56–80 age group, lateral occipital, postcentral and precuneus also showed significantly reduced cortical thickness at 3 T compared to 1.5 T for both genders. Also a larger number of regions showed significant differences between 1.5 T and 3 T in females than in males. Tables 5–10 summarize these field strength differences for different age groups.

**Table 5:** Adjusted means and mean differences (95% confidence interval) of regional cortical thickness between 1.5 T and 3 T for females in the age group 20–40 after controlling for confounding effects.

Region						
	Left Hemisphere			Right Hemisphere		
	1.5 T	3 T	Mean differences	1.5 T	3 T	Mean differences
Banks of the STS^+^	2.52	2.58	0.07 (−0.03, 0.17)	2.46*	2.62*	0.16 (0.07, 0.25)
Caudal anterior cingulate	2.61*	2.75*	0.14 (0.01, 0.27)	2.76	2.89	0.13 (0.00, 0.27)
Caudal middle frontal	2.52	2.60	0.08 (0.00, 0.17)	2.51	2.57	0.06 (−0.03, 0.15)
Cuneus	1.89	1.89	0.00 (−0.07, 0.07)	1.87	1.82	−0.05 (−0.13, 0.03)
Entorhinal	3.11	3.14	0.04 (−0.16, 0.23)	2.94	3.04	0.11 (−0.09, 0.30)
Fusiform	2.55*	2.70*	0.15 (0.07, 0.24)	2.54*	2.65*	0.11 (0.03, 0.20)
Inferior parietal	2.46*	2.54*	0.08 (0.00, 0.16)	2.52	2.58	0.06 (−0.02, 0.14)
Inferior temporal	2.74	2.78	0.04 (−0.05, 0.12)	2.73*	2.86*	0.13 (0.04, 0.22)
Isthmus cingulate	2.51*	2.63*	0.12 (0.01, 0.23)	2.44	2.50	0.05 (−0.05, 0.16)
Lateral occipital	2.20	2.22	0.02 (−0.06, 0.10)	2.31	2.29	−0.03 (−0.11, 0.06)
Lateral oribitofrontal	2.64*	2.77*	0.13 (0.03, 0.22)	2.74	2.77	0.04 (−0.05, 0.13)
Lingual	1.98	2.04	0.06 (−0.01, 0.14)	1.96	1.90	−0.06 (−0.13, 0.02)
Medial orbitofrontal	2.59	2.60	0.01 (−0.08, 0.10)	2.85	2.88	0.04 (−0.06, 0.13)
Middle temporal	2.80	2.84	0.04 (−0.05, 0.14)	2.74*	2.85*	0.10 (0.01, 0.19)
parahippocampal	2.58*	2.88*	0.31 (0.13, 0.48)	2.44*	2.73*	0.30 (0.14, 0.46)
paracentral	2.25*	2.37*	0.12 (0.02, 0.22)	2.11*	2.23*	0.12 (0.03, 0.22)
Pars opercularis	2.61	2.69	0.08 (−0.01, 0.17)	2.67	2.71	0.04 (−0.05, 0.13)
Pars orbitalis	2.68	2.74	0.06 (−0.06, 0.19)	2.70	2.67	−0.03 (−0.17, 0.11)
Pars triangularis	2.50	2.56	0.06 (−0.04, 0.16)	2.54	2.56	0.03 (−0.07, 0.13)
Pericalcarine	1.58	1.64	0.06 (−0.02, 0.13)	1.53	1.48	−0.06 (−0.14, 0.02)
Postcentral	2.06	2.11	0.04 (−0.03, 0.12)	1.98*	2.08*	0.10 (0.03, 0.17)
Posterior cingulate	2.49*	2.62*	0.13 (0.04, 0.22)	2.46*	2.56*	0.10 (0.00, 0.19)
Precentral	2.43*	2.54*	0.11 (0.01, 0.20)	2.27*	2.38*	0.12 (0.02, 0.21)
Precuneus	2.34*	2.44*	0.10 (0.02, 0.18)	2.28*	2.36*	0.08 (0.00, 0.16)
Rostral anterior cingulate	2.93*	3.10*	0.17 (0.04, 0.30)	3.12	3.22	0.10 (−0.04, 0.24)
Rostral middle frontal	2.46	2.41	−0.05 (−0.12, 0.03)	2.52	2.52	0.00 (−0.08, 0.08)
Superior frontal	2.77	2.83	0.06 (−0.03, 0.16)	2.80	2.87	0.07 (−0.02, 0.16)
Superior parietal	2.16	2.23	0.07 (−0.01, 0.14)	2.15	2.23	0.08 (0.00, 0.16)
Superior temporal	2.72*	2.83*	0.11 (0.01, 0.20)	2.66*	2.77*	0.12 (0.02, 0.22)
Supramarginal	2.54	2.60	0.06 (−0.02, 0.14)	2.55*	2.64*	0.09 (0.01, 0.17)
Frontal pole	2.88	2.88	−0.01 (−0.17, 0.15)	3.01	2.99	−0.02 (−0.17, 0.14)
Temporal pole	3.44	3.64	0.20 (−0.01, 0.41)	3.59	3.64	0.05 (−0.15, 0.25)
Transverse temporal	2.33	2.42	0.09 (−0.04, 0.22)	2.17*	2.34*	0.18 (0.04, 0.32)
Insula	3.01*	3.11*	0.10 (0.01, 0.19)	3.11	3.13	0.02 (−0.08, 0.11)

Note: overall field strength effect on regional cortical thickness is significant with p-value < 0.0001 using Pillai's Trace test; Factors ajusted for include: age, gender, resolution, scanner type, sequence, and interactions of field*age, and field*gender. ^+^STS  =  superior temporal sulcus.

**Table 6:** Adjusted means and mean differences (95% confidence interval) of regional cortical thickness between 1.5 T and 3 T for females in the age group 41–55 after controlling for confounding effects.

Region						
	Left Hemisphere			Right Hemisphere		
	1.5 T	3 T	Mean differences	1.5 T	3 T	Mean differences
Banks of the STS^+^	2.52	2.54	0.02 (−0.11, 0.16)	2.53	2.57	0.04 (−0.09, 0.17)
Caudal anterior cingulate	2.62	2.72	0.10 (−0.08, 0.28)	2.77	2.80	0.04 (−0.14, 0.22)
Caudal middle frontal	2.56	2.52	−0.04 (−0.16, 0.08)	2.57	2.46	−0.11 (−0.23, 0.01)
Cuneus	1.91	1.83	−0.08 (−0.18, 0.02)	1.87	1.77	−0.10 (−0.21, 0.01)
Entorhinal	3.14	3.24	0.10 (−0.16, 0.36)	3.01	2.94	−0.06 (−0.33, 0.20)
Fusiform	2.52	2.62	0.10 (−0.02, 0.21)	2.48	2.59	0.11 (0.00, 0.22)
Inferior parietal	2.46	2.53	0.06 (−0.05, 0.17)	2.53	2.54	0.01 (−0.09, 0.12)
Inferior temporal	2.72	2.73	0.01 (−0.11, 0.12)	2.72	2.78	0.06 (−0.07, 0.18)
Isthmus cingulate	2.53	2.45	−0.08 (−0.23, 0.07)	2.52	2.40	−0.13 (−0.27, 0.02)
Lateral occipital	2.21	2.20	−0.02 (−0.13, 0.09)	2.34	2.29	−0.05 (−0.15, 0.06)
Lateral oribitofrontal	2.63	2.65	0.03 (−0.10, 0.15)	2.74	2.65	−0.09 (−0.21, 0.03)
Lingual	1.95	1.98	0.03 (−0.07, 0.13)	2.01*	1.88*	−0.13 (−0.23, −0.02)
Medial orbitofrontal	2.51	2.49	−0.02 (−0.15, 0.10)	2.83	2.78	−0.05 (−0.18, 0.08)
Middle temporal	2.82	2.80	−0.02 (−0.15, 0.11)	2.76	2.77	0.01 (−0.11, 0.14)
parahippocampal	2.48	2.66	0.18 (−0.05, 0.42)	2.39	2.56	0.17 (−0.05, 0.39)
paracentral	2.28	2.23	−0.05 (−0.19, 0.08)	2.09	2.14	0.04 (−0.08, 0.17)
Pars opercularis	2.62	2.60	−0.02 (−0.14, 0.10)	2.69	2.63	−0.06 (−0.18, 0.07)
Pars orbitalis	2.67	2.62	−0.05 (−0.22, 0.12)	2.68	2.50	−0.18 (−0.37, 0.01)
Pars triangularis	2.49	2.48	−0.01 (−0.14, 0.13)	2.55	2.48	−0.06 (−0.20, 0.07)
Pericalcarine	1.58	1.60	0.02 (−0.08, 0.12)	1.56*	1.44*	−0.12 (−0.23, −0.01)
Postcentral	2.08	2.08	0.00 (−0.10, 0.10)	1.97	2.04	0.07 (−0.03, 0.16)
Posterior cingulate	2.46	2.48	0.02 (−0.10, 0.14)	2.49	2.51	0.03 (−0.10, 0.15)
Precentral	2.50	2.42	−0.08 (−0.21, 0.05)	2.33	2.26	−0.07 (−0.20, 0.06)
Precuneus	2.33	2.33	−0.01 (−0.11, 0.10)	2.25	2.33	0.08 (−0.03, 0.18)
Rostral anterior cingulate	2.86	2.93	0.08 (−0.10, 0.25)	3.05	3.13	0.09 (−0.11, 0.28)
Rostral middle frontal	2.46*	2.35*	−0.11 (−0.22, −0.01)	2.52	2.45	−0.07 (−0.19, 0.04)
Superior frontal	2.78	2.70	−0.08 (−0.21, 0.05)	2.84	2.74	−0.09 (−0.22, 0.03)
Superior parietal	2.17	2.18	0.00 (−0.10, 0.11)	2.15	2.18	0.03 (−0.08, 0.14)
Superior temporal	2.71	2.74	0.03 (−0.10, 0.15)	2.64	2.68	0.04 (−0.09, 0.18)
Supramarginal	2.58	2.56	−0.02 (−0.13, 0.09)	2.56	2.58	0.01 (−0.10, 0.12)
Frontal pole	2.84	2.73	−0.11 (−0.33, 0.11)	3.12	3.01	−0.11 (−0.33, 0.10)
Temporal pole	3.44	3.39	−0.05 (−0.34, 0.23)	3.55	3.50	−0.05 (−0.33, 0.22)
Transverse temporal	2.36	2.29	−0.07 (−0.25, 0.10)	2.18	2.13	−0.05 (−0.25, 0.14)
Insula	2.97	2.97	0.00 (−0.12, 0.12)	3.08	3.06	−0.02 (−0.15, 0.11)

Note: overall field strength effect on regional cortical thickness is significant with p-value <0.0001 using Pillai's Trace test; Factors ajusted for include: age, gender, resolution, scanner type, sequence, and interactions of field*age, and field*gender. **^+^**STS  =  superior temporal sulcus.

**Table 7:** Adjusted means and mean differences (95% confidence interval) of regional cortical thickness between 1.5 T and 3 T for females in the age group 56–80 after controlling for confounding effects.

Region						
	Left Hemisphere			Right Hemisphere		
	1.5 T	3 T	Mean differences	1.5 T	3 T	Mean differences
Banks of the STS^+^	2.49	2.49	−0.01 (−0.15, 0.14)	2.48	2.52	0.04 (−0.10, 0.17)
Caudal anterior cingulate	2.66	2.62	−0.04 (−0.23, 0.15)	2.78	2.76	−0.02 (−0.21, 0.17)
Caudal middle frontal	2.57	2.51	−0.06 (−0.18, 0.07)	2.55	2.48	−0.07 (−0.20, 0.06)
Cuneus	1.95*	1.78*	−0.17 (−0.28, −0.07)	1.94*	1.72*	−0.22 (−0.33, −0.11)
Entorhinal	3.30	3.12	−0.18 (−0.45, 0.09)	3.09	2.94	−0.15 (−0.43, 0.12)
Fusiform	2.59	2.57	−0.02 (−0.14, 0.10)	2.51	2.52	0.01 (−0.10, 0.13)
Inferior parietal	2.49	2.48	−0.01 (−0.12, 0.11)	2.56	2.48	−0.07 (−0.18, 0.04)
Inferior temporal	2.75	2.69	−0.05 (−0.18, 0.07)	2.71	2.81	0.09 (−0.03, 0.22)
Isthmus cingulate	2.56	2.41	−0.15 (−0.30, 0.01)	2.55*	2.32*	−0.23 (−0.39, −0.08)
Lateral occipital	2.24	2.16	−0.08 (−0.19, 0.04)	2.38*	2.23*	−0.14 (−0.26, −0.03)
Lateral oribitofrontal	2.55*	2.68*	0.13 (0.00, 0.26)	2.70	2.67	−0.03 (−0.15, 0.09)
Lingual	2.00	1.91	−0.09 (−0.19, 0.01)	2.06*	1.80*	−0.27 (−0.37, −0.16)
Medial orbitofrontal	2.49	2.46	−0.02 (−0.15, 0.11)	2.81	2.78	−0.03 (−0.17, 0.11)
Middle temporal	2.77	2.79	0.02 (−0.11, 0.15)	2.74	2.75	0.01 (−0.12, 0.13)
parahippocampal	2.62	2.55	−0.07 (−0.31, 0.18)	2.47	2.50	0.03 (−0.20, 0.26)
paracentral	2.32	2.20	−0.12 (−0.26, 0.02)	2.14	2.07	−0.07 (−0.21, 0.06)
Pars opercularis	2.65	2.54	−0.11 (−0.24, 0.01)	2.68	2.56	−0.12 (−0.25, 0.01)
Pars orbitalis	2.64	2.65	0.01 (−0.17, 0.19)	2.69	2.56	−0.12 (−0.32, 0.07)
Pars triangularis	2.51	2.46	−0.05 (−0.19, 0.09)	2.57*	2.39*	−0.18 (−0.32, −0.04)
Pericalcarine	1.61	1.57	−0.04 (−0.15, 0.06)	1.62*	1.40*	−0.22 (−0.33, −0.11)
Postcentral	2.09	2.03	−0.06 (−0.16, 0.05)	1.97	2.04	0.07 (−0.03, 0.17)
Posterior cingulate	2.50	2.45	−0.05 (−0.18, 0.07)	2.46	2.44	−0.03 (−0.16, 0.11)
Precentral	2.59*	2.37*	−0.23 (−0.36, −0.09)	2.38*	2.21*	−0.17 (−0.30, −0.03)
Precuneus	2.35	2.30	−0.05 (−0.16, 0.07)	2.27	2.27	0.00 (−0.11, 0.11)
Rostral anterior cingulate	2.81	2.82	0.02 (−0.17, 0.20)	3.04	3.04	0.00 (−0.20, 0.20)
Rostral middle frontal	2.45*	2.33*	−0.12 (−0.23, −0.01)	2.51	2.41	−0.10 (−0.21, 0.02)
Superior frontal	2.80	2.68	−0.12 (−0.26, 0.01)	2.85*	2.72*	−0.13 (−0.26, 0.00)
Superior parietal	2.19	2.17	−0.02 (−0.13, 0.09)	2.20	2.18	−0.02 (−0.14, 0.09)
Superior temporal	2.73	2.65	−0.08 (−0.21, 0.05)	2.64	2.59	−0.06 (−0.20, 0.08)
Supramarginal	2.61	2.50	−0.11 (−0.22, 0.01)	2.57	2.51	−0.05 (−0.17, 0.06)
Frontal pole	2.86	2.70	−0.16 (−0.39, 0.07)	2.99	2.94	−0.06 (−0.28, 0.17)
Temporal pole	3.52	3.34	−0.18 (−0.48, 0.12)	3.57	3.46	−0.11 (−0.39, 0.18)
Transverse temporal	2.41	2.27	−0.14 (−0.33, 0.04)	2.24	2.13	−0.11 (−0.31, 0.09)
Insula	2.98	2.93	−0.05 (−0.18, 0.08)	3.11	2.99	−0.12 (−0.26, 0.01)

Note: overall field strength effect on regional cortical thickness is significant with p-value <0.0001 using Pillai's Trace test; Factors ajusted for include: age, gender, resolution, scanner type, sequence, and interactions of field*age, and field*gender. **^+^**STS  =  superior temporal sulcus.

**Table 8:** Adjusted means and mean differences (95% confidence interval) of regional cortical thickness between 1.5 T and 3 T for males in the age group 20–40 after controlling for confounding effects.

Region						
	Left Hemisphere			Right Hemisphere		
	1.5 T	3 T	Mean differences	1.5 T	3 T	Mean differences
Banks of the STS^+^	2.56	2.61	0.05 (−0.04, 0.14)	2.49*	2.63*	0.14 (0.05, 0.22)
Caudal anterior cingulate	2.62	2.72	0.10 (−0.02, 0.22)	2.79	2.79	0.00 (−0.12, 0.12)
Caudal middle frontal	2.53	2.56	0.03 (−0.05, 0.11)	2.49	2.53	0.03 (−0.05, 0.11)
Cuneus	1.86	1.86	0.00 (−0.06, 0.07)	1.81	1.79	−0.02 (−0.09, 0.05)
Entorhinal	3.09*	3.27*	0.18 (0.00, 0.35)	2.79*	3.06*	0.27 (0.10, 0.45)
Fusiform	2.58*	2.72*	0.13 (0.06, 0.21)	2.56*	2.65*	0.09 (0.01, 0.16)
Inferior parietal	2.48	2.55	0.07 (0.00, 0.14)	2.52	2.59	0.07 (0.00, 0.14)
Inferior temporal	2.79	2.86	0.07 (−0.01, 0.14)	2.85	2.92	0.07 (−0.02, 0.15)
Isthmus cingulate	2.46*	2.57*	0.12 (0.02, 0.21)	2.34*	2.46*	0.12 (0.03, 0.22)
Lateral occipital	2.20	2.21	0.02 (−0.05, 0.09)	2.27	2.31	0.04 (−0.03, 0.11)
Lateral oribitofrontal	2.73	2.73	0.00 (−0.08, 0.09)	2.79	2.78	−0.01 (−0.08, 0.07)
Lingual	1.99	2.03	0.04 (−0.03, 0.10)	1.90	1.91	0.01 (−0.06, 0.08)
Medial orbitofrontal	2.69*	2.57*	−0.12 (−0.21, −0.04)	2.92	2.91	0.00 (−0.09, 0.08)
Middle temporal	2.85	2.91	0.07 (−0.02, 0.15)	2.80	2.86	0.06 (−0.02, 0.14)
parahippocampal	2.58*	2.81*	0.23 (0.07, 0.38)	2.39*	2.64*	0.25 (0.10, 0.39)
paracentral	2.21*	2.32*	0.11 (0.02, 0.20)	2.11	2.15	0.04 (−0.05, 0.12)
Pars opercularis	2.63	2.68	0.05 (−0.03, 0.13)	2.67	2.68	0.00 (−0.08, 0.09)
Pars orbitalis	2.74	2.72	−0.02 (−0.13, 0.09)	2.70	2.61	−0.09 (−0.22, 0.03)
Pars triangularis	2.56	2.55	0.00 (−0.09, 0.08)	2.53	2.52	−0.01 (−0.10, 0.08)
Pericalcarine	1.61	1.63	0.01 (−0.05, 0.08)	1.52	1.44	−0.07 (−0.14, 0.00)
Postcentral	2.06	2.08	0.02 (−0.05, 0.09)	2.04	2.02	−0.02 (−0.08, 0.05)
Posterior cingulate	2.50*	2.61*	0.11 (0.03, 0.19)	2.47	2.53	0.06 (−0.02, 0.15)
Precentral	2.32*	2.48*	0.17 (0.08, 0.25)	2.17*	2.31*	0.14 (0.06, 0.23)
Precuneus	2.37	2.41	0.04 (−0.03, 0.11)	2.29	2.33	0.04 (−0.03, 0.11)
Rostral anterior cingulate	3.03	3.06	0.03 (−0.09, 0.15)	3.22	3.22	0.00 (−0.13, 0.12)
Rostral middle frontal	2.49*	2.40*	−0.08 (−0.15, −0.01)	2.56	2.50	−0.06 (−0.14, 0.01)
Superior frontal	2.76	2.76	0.00 (−0.08, 0.09)	2.80	2.81	0.01 (−0.07, 0.09)
Superior parietal	2.17	2.19	0.02 (−0.05, 0.09)	2.18	2.20	0.02 (−0.05, 0.09)
Superior temporal	2.76	2.81	0.05 (−0.04, 0.13)	2.69*	2.79*	0.10 (0.01, 0.19)
Supramarginal	2.53	2.59	0.06 (−0.01, 0.14)	2.55	2.60	0.05 (−0.03, 0.12)
Frontal pole	2.92	2.84	−0.08 (−0.23, 0.07)	3.08	3.04	−0.04 (−0.18, 0.10)
Temporal pole	3.46	3.64	0.18 (0.00, 0.37)	3.65	3.65	0.00 (−0.18, 0.18)
Transverse temporal	2.26	2.32	0.06 (−0.05, 0.18)	2.08*	2.25*	0.17 (0.05, 0.30)
Insula	3.09	3.10	0.00 (−0.08, 0.09)	3.16	3.12	−0.04 (−0.13, 0.05)

Note: overall field strength effect on regional cortical thickness is significant with p-value <0.0001 using Pillai's Trace test; Factors ajusted for include: age, gender, resolution, scanner type, sequence, and interactions of field*age, and field*gender. **^+^**STS  =  superior temporal sulcus.

**Table 9:** Adjusted means and mean differences (95% confidence interval) of regional cortical thickness between 1.5 T and 3 T for males in the age group 41–55 after controlling for confounding effects.

Region						
	Left Hemisphere			Right Hemisphere		
	1.5 T	3 T	Mean differences	1.5 T	3 T	Mean differences
Banks of the STS^+^	2.56	2.57	0.00 (−0.13, 0.14)	2.57	2.58	0.01 (−0.11, 0.14)
Caudal anterior cingulate	2.64	2.69	0.06 (−0.12, 0.23)	2.68	2.58	−0.10 (−0.28, 0.08)
Caudal middle frontal	2.57	2.48	−0.09 (−0.21, 0.03)	2.57*	2.43*	−0.14 (−0.26, −0.02)
Cuneus	1.88	1.81	−0.08 (−0.18, 0.02)	1.85	1.78	−0.07 (−0.18, 0.03)
Entorhinal	3.12	3.36	0.24 (−0.01, 0.50)	3.07	3.18	0.10 (−0.16, 0.36)
Fusiform	2.56	2.64	0.08 (−0.03, 0.19)	2.51	2.59	0.08 (−0.03, 0.19)
Inferior parietal	2.49	2.54	0.05 (−0.06, 0.16)	2.50	2.52	0.02 (−0.08, 0.12)
Inferior temporal	2.78	2.81	0.03 (−0.08, 0.15)	2.79	2.78	−0.01 (−0.13, 0.11)
Isthmus cingulate	2.48	2.40	−0.08 (−0.23, 0.06)	2.44	2.38	−0.06 (−0.20, 0.08)
Lateral occipital	2.21	2.19	−0.02 (−0.13, 0.08)	2.22	2.24	0.02 (−0.09, 0.13)
Lateral oribitofrontal	2.72	2.62	−0.10 (−0.22, 0.03)	2.76*	2.63*	−0.13 (−0.25, −0.02)
Lingual	1.97	1.97	0.00 (−0.09, 0.10)	2.01	1.95	−0.06 (−0.16, 0.04)
Medial orbitofrontal	2.61*	2.46*	−0.15 (−0.28, −0.03)	2.63	2.54	−0.09 (−0.21, 0.04)
Middle temporal	2.87	2.87	0.00 (−0.12, 0.13)	2.87	2.84	−0.03 (−0.15, 0.09)
parahippocampal	2.48	2.58	0.10 (−0.13, 0.33)	2.41	2.54	0.12 (−0.09, 0.34)
paracentral	2.25	2.19	−0.06 (−0.19, 0.07)	2.21	2.17	−0.04 (−0.17, 0.08)
Pars opercularis	2.64	2.59	−0.05 (−0.17, 0.07)	2.66	2.57	−0.09 (−0.22, 0.03)
Pars orbitalis	2.73	2.59	−0.14 (−0.30, 0.03)	2.72*	2.47*	−0.24 (−0.43, −0.06)
Pars triangularis	2.55	2.47	−0.07 (−0.20, 0.06)	2.55	2.45	−0.10 (−0.23, 0.03)
Pericalcarine	1.62	1.59	−0.03 (−0.12, 0.07)	1.63*	1.50*	−0.13 (−0.24, −0.03)
Postcentral	2.07	2.06	−0.02 (−0.11, 0.08)	2.05	2.00	−0.05 (−0.15, 0.05)
Posterior cingulate	2.47	2.47	0.01 (−0.11, 0.12)	2.51	2.51	−0.01 (−0.13, 0.11)
Precentral	2.39	2.37	−0.02 (−0.15, 0.11)	2.36	2.32	−0.04 (−0.17, 0.08)
Precuneus	2.36	2.29	−0.07 (−0.17, 0.04)	2.32	2.35	0.03 (−0.08, 0.14)
Rostral anterior cingulate	2.96	2.90	−0.06 (−0.24, 0.11)	2.96	2.94	−0.02 (−0.21, 0.17)
Rostral middle frontal	2.49*	2.34*	−0.15 (−0.26, −0.05)	2.48*	2.34*	−0.14 (−0.25, −0.03)
Superior frontal	2.76*	2.62*	−0.14 (−0.26, −0.02)	2.78*	2.63*	−0.15 (−0.27, −0.03)
Superior parietal	2.18	2.14	−0.04 (−0.15, 0.06)	2.19	2.17	−0.02 (−0.13, 0.08)
Superior temporal	2.76	2.73	−0.03 (−0.16, 0.09)	2.74	2.77	0.02 (−0.11, 0.15)
Supramarginal	2.57	2.55	−0.02 (−0.13, 0.09)	2.59	2.56	−0.03 (−0.14, 0.07)
Frontal pole	2.87	2.69	−0.18 (−0.40, 0.03)	2.95	2.81	−0.13 (−0.34, 0.08)
Temporal pole	3.46	3.39	−0.07 (−0.35, 0.21)	3.62	3.52	−0.10 (−0.37, 0.17)
Transverse temporal	2.28	2.18	−0.10 (−0.27, 0.07)	2.25	2.19	−0.06 (−0.24, 0.13)
Insula	3.05	2.96	−0.10 (−0.22, 0.03)	3.05	2.97	−0.07 (−0.20, 0.05)

Note: overall field strength effect on regional cortical thickness is significant with p-value <0.0001 using Pillai's Trace test; Factors ajusted for include: age, gender, resolution, scanner type, sequence, and interactions of field*age, and field*gender. ^+^STS  =  superior temporal sulcus.

**Table 10:** Adjusted means and mean differences (95% confidence interval) of regional cortical thickness between 1.5 T and 3 T for males in the age group 56–80 after controlling for confounding effects.

Region						
	Left Hemisphere			Right Hemisphere		
	1.5 T	3 T	Mean differences	1.5 T	3 T	Mean differences
Banks of the STS^+^	2.54	2.51	−0.02 (−0.15, 0.10)	2.52	2.53	0.01 (−0.11, 0.14)
Caudal anterior cingulate	2.68	2.59	−0.08 (−0.25, 0.09)	2.69	2.53	−0.16 (−0.33, 0.02)
Caudal middle frontal	2.58	2.47	−0.11 (−0.23, 0.00)	2.55	2.45	−0.10 (−0.21, 0.02)
Cuneus	1.92*	1.75*	−0.17 (−0.27, −0.07)	1.92*	1.73*	−0.19 (−0.30, −0.09)
Entorhinal	3.28	3.24	−0.04 (−0.29, 0.21)	3.16	3.17	0.01 (−0.24, 0.27)
Fusiform	2.62	2.59	−0.03 (−0.14, 0.08)	2.54	2.53	−0.01 (−0.12, 0.09)
Inferior parietal	2.51	2.49	−0.02 (−0.13, 0.08)	2.53	2.47	−0.07 (−0.17, 0.04)
Inferior temporal	2.80	2.77	−0.02 (−0.14, 0.09)	2.79	2.81	0.03 (−0.09, 0.14)
Isthmus cingulate	2.51*	2.36*	−0.15 (−0.29, −0.01)	2.47*	2.30*	−0.16 (−0.30, −0.02)
Lateral occipital	2.23	2.15	−0.08 (−0.18, 0.02)	2.26	2.18	−0.08 (−0.18, 0.03)
Lateral oribitofrontal	2.64	2.65	0.01 (−0.11, 0.13)	2.72	2.64	−0.07 (−0.19, 0.04)
Lingual	2.01*	1.89*	−0.12 (−0.21, −0.02)	2.06*	1.87*	−0.20 (−0.30, −0.10)
Medial orbitofrontal	2.59*	2.43*	−0.15 (−0.27, −0.04)	2.60	2.53	−0.07 (−0.19, 0.05)
Middle temporal	2.82	2.86	0.04 (−0.08, 0.16)	2.86	2.82	−0.04 (−0.15, 0.08)
parahippocampal	2.62	2.47	−0.15 (−0.37, 0.08)	2.50	2.48	−0.02 (−0.22, 0.19)
paracentral	2.29*	2.15*	−0.13 (−0.26, −0.01)	2.26*	2.10*	−0.16 (−0.28, −0.04)
Pars opercularis	2.67*	2.53*	−0.14 (−0.25, −0.03)	2.65*	2.50*	−0.16 (−0.28, −0.04)
Pars orbitalis	2.70	2.62	−0.07 (−0.24, 0.09)	2.73*	2.54*	−0.19 (−0.37, −0.01)
Pars triangularis	2.56	2.45	−0.11 (−0.24, 0.01)	2.57*	2.36*	−0.21 (−0.34, −0.09)
Pericalcarine	1.65	1.56	−0.09 (−0.18, 0.01)	1.70*	1.47*	−0.23 (−0.34, −0.13)
Postcentral	2.09	2.01	−0.08 (−0.17, 0.01)	2.05	2.01	−0.04 (−0.14, 0.05)
Posterior cingulate	2.51	2.44	−0.07 (−0.19, 0.05)	2.49	2.43	−0.06 (−0.18, 0.06)
Precentral	2.48*	2.31*	−0.17 (−0.29, −0.05)	2.42*	2.28*	−0.14 (−0.26, −0.02)
Precuneus	2.38*	2.27*	−0.11 (−0.21, −0.01)	2.33	2.29	−0.05 (−0.15, 0.06)
Rostral anterior cingulate	2.91	2.79	−0.12 (−0.29, 0.05)	2.96	2.85	−0.11 (−0.29, 0.08)
Rostral middle frontal	2.48*	2.32*	−0.16 (−0.26, −0.06)	2.46*	2.31*	−0.16 (−0.27, −0.05)
Superior frontal	2.79*	2.60*	−0.19 (−0.31, −0.06)	2.79*	2.60*	−0.19 (−0.31, −0.07)
Superior parietal	2.20	2.13	−0.07 (−0.17, 0.04)	2.24	2.17	−0.08 (−0.18, 0.03)
Superior temporal	2.78*	2.64*	−0.14 (−0.26, −0.02)	2.74	2.67	−0.08 (−0.20, 0.05)
Supramarginal	2.59	2.48	−0.11 (−0.21, 0.00)	2.59	2.49	−0.10 (−0.20, 0.01)
Frontal pole	2.89*	2.67*	−0.23 (−0.44, −0.02)	2.81	2.74	−0.07 (−0.28, 0.13)
Temporal pole	3.54	3.34	−0.20 (−0.47, 0.07)	3.64	3.49	−0.15 (−0.41, 0.10)
Transverse temporal	2.33*	2.16*	−0.17 (−0.34, −0.01)	2.31	2.20	−0.11 (−0.29, 0.07)
Insula	3.07*	2.92*	−0.15 (−0.26, −0.03)	3.08*	2.90*	−0.18 (−0.31, −0.06)

Note: overall field strength effect on regional cortical thickness is significant with p-value <0.0001 using Pillai's Trace test; Factors ajusted for include: age, gender, resolution, scanner type, sequence, and interactions of field*age, and field*gender. **^+^**STS  =  superior temporal sulcus.

**Figure 4:**
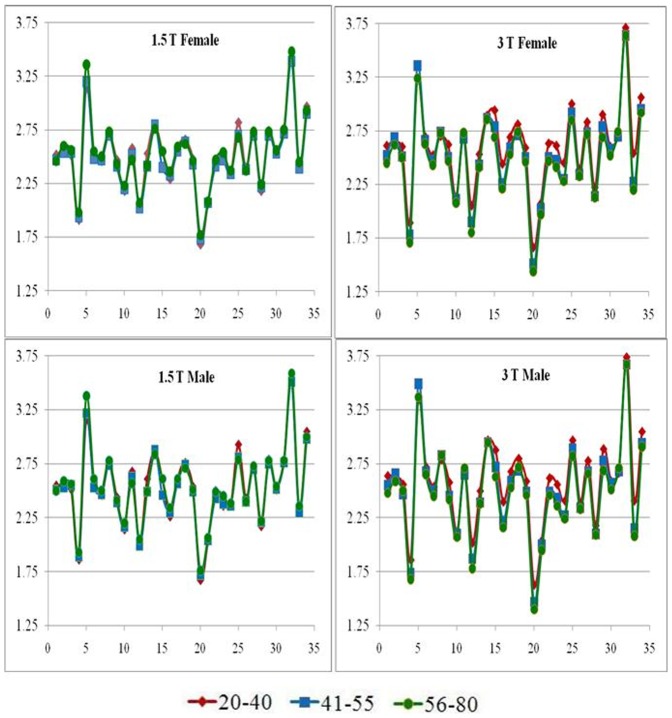
Regional cortical thickness of the 34 segmented regions plotted for the three age groups.

**Figure 5:**
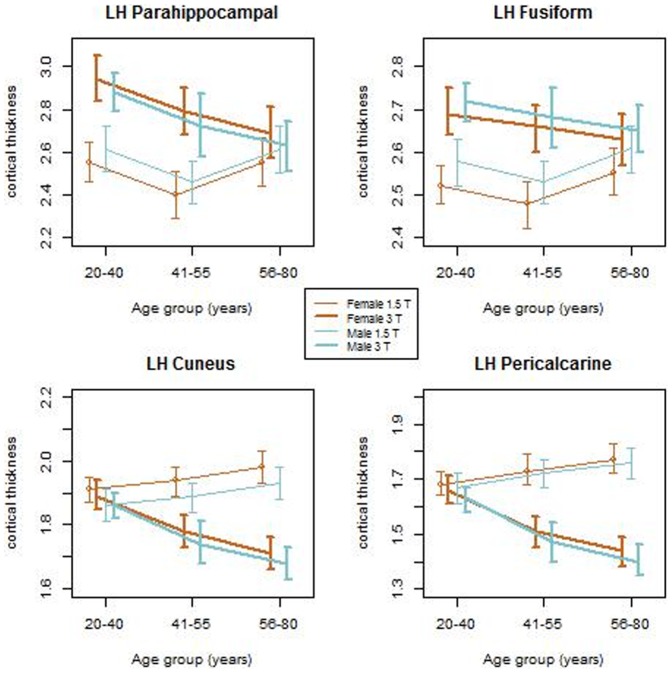
An example of the variation of cortical thickness for three age groups for males and females at 1.5 T and 3 T. The following regions in left hemisphere are shown in this plat: parahippocampal, fusiform, cuneus and pericalcarine

For a better visualization of the above described effects of field strength on cortical thickness for both genders at the three age groups, we plotted the mean regional cortical thicknesses for the 34 segmented regions (Fig. 4). The plots show that the differences in cortical thickness with age are very subtle and are only apparent at 3 T. The figure also shows that these subtle differences in cortical thickness follow similar patterns in both male and female subjects. Further, we explored the age related changes in the mean regional cortical thickness for the two genders at the three different age groups. As an example, Fig. 5 shows these changes in parahippocampal, fusiform, cuneus and pericalcarine. From these plots, it can be seen that the male and female groups follow the same progression across the three age groups. However, the differences in cortical thickness between 1.5 T and 3 T were observed to be significant. For the regions where the cortical thickness was higher at 3 T than at 1.5 T, the effect was higher for the younger age group and started to diminish with age, whereas for the regions where the thickness is higher at 1.5 T than at 3 T, the effect is higher in the older age groups. These results suggest that the field strength dependence is independent of the gender.

## Discussion

The primary purpose of this study was to perform a comprehensive analysis of the effect of field strength on the global and regional cortical thicknesses and explore how gender and age further affect these field strength differences in a relatively large normal cohort. In order to accomplish this objective, we pooled data from various publicly available databases. We paid attention to balance the age, gender and field strength among different sub cohorts. Great care was taken to eliminate images with incidental pathology, images with poor signal-to-noise ratio and artifacts. In order to specifically evaluate the effect of field strength, the statistical analysis included the other extraneous factors such as age, gender, pulse sequence, scanner type and image resolution as covariates. We implemented sophisticated statistical analysis methods to realize the objective of this study. We believe that this is the first study that investigated the effect of magnetic field strength on global and regional cortical thicknesses in a relatively large cohort of normal controls.

Ideally the measured cortical thickness should be independent of extrinsic factors such as field strength, pulse sequence, and scanner platform. However, in practice the estimated cortical thickness using FreeSurfer or any other software package is influenced by the spatial resolution and contrast-to-noise ratio (CNR) in the images. It is well known that both spatial resolution and CNR are higher at 3T compared to 1.5T. This improvement in turn should lead to better WM surface and pial surface reconstruction and improved accuracy in the estimation of cortical thickness.

### Effect of gender

Our results indicate that neither field strength nor gender has an effect on the global cortical thickness. Our results also indicate that field strength has a significant effect on the measured regional cortical thickness and that these differences seem to be influenced by gender. Our results show that at 1.5 T, various regions show higher cortical thickness in females than in males (Fig. 4 and Fig. 5) whereas at 3 T, these differences do not seem to be significant. There is significant literature suggesting cortical thickness differences between males and females in both normal and diseased populations [1], [3], [5], [6], [8]. All these studies were conducted at 1.5 T and our results also suggests similar differences at 1.5 T. But our results at 3 T show no significant differences and that seems to suggest that the gender-based differences in cortical thickness may be an artifact arising from extrinsic factors such as field strength rather than intrinsic.

### Effect of age

We also investigated if the field strength based differences in cortical thickness are influenced by age. In the regions that show higher cortical thickness at 3 T compared to 1.5 T (parahippocampal, fusiform), the differences seem to be more prominent in the younger age group (20–40). However, in regions that showed higher cortical thickness at 1.5 T compared to 3 T (pericalcarine, cuneus), the differences seem to originate from the older age groups (41–55 and 56–80). Furthermore, our results from the 3 T data suggest that cortical thickness in all regions steadily decreases with age for both genders. From our results, it seems likely that in the traditionally thinner cortical regions such as the cuneus and pericalcarine and also other regions where cortical thickness decreases with age, the data from 1.5 T does not have the necessary CNR and resolution to correctly segment out the pial and white surfaces. Because of this, there may be an inaccurate estimation of cortical thickness in those regions at 1.5 T. This perhaps explains the observed differences in the age-based trend of cortical thickness at 3 T and 1.5 T (Fig. 5). These results once again suggest that field strength has considerable influence on the measured cortical thickness.

### Comparison with published studies

Han et al [11] studied the effect of field strength on the cortical thickness in a small sample of 15 subjects and reported higher global mean cortical thickness at 3 T relative to 1.5 T. Our results in this large cohort do not support this. For regional cortical thickness measurements, Han et al [11] showed that the difference is up to 0.2 mm across different cortical regions. This is consistent with our results which show up to 0.25 mm thicker cortex at 3 T compared to 1.5 T. They also reported a similar pattern of regional differences across field strengths. The limitations of the study by Han et al [11] are, however, the small sample size and narrow age distribution (66–81 years). These authors did not investigate the effects of age and gender. In another study, Wonderlick et al [12] evaluated the effect of image resolution, parallel acquisition techniques and pulse sequence on volume and cortical thickness measurements and concluded that these parameters did not affect reliability of cortical thickness measurement. Our statistical analyses agree with these results since we also found that neither image resolution nor pulse sequence had any effect on cortical thickness measurement even on a large cohort of subjects. However, the limitation of their study is small sample size of 11 subjects scanned at a single field strength of 3 T.

### Variability in regional cortical thickness

In a volume-based study using FreeSurfer's cortical parcellation tools and comparing 1.5 T and 3 T data, Pfefferbaum et al [25] found higher differences in regions such as the precentral gyrus, and the occipital cortex. Other reliability studies found evidence of non-uniform variability in cortical thickness measurements when comparing different processing conditions such as workstation or FreeSurfer version [26] and highly significant differences were reported predominantly in the frontal and temporal cortices. Differences in other regional thicknesses may be due to the immediate proximity of other brain structures such as blood vessels, dura and hippocampus impacting the accuracy of the cortical segmentation. This is especially true for the medial frontal area, anterior temporal regions [11].

Although the MRI-derived cortical thickness measurements based on FreeSurfer have been previously validated against manual segmentation on brain scans acquired both in vivo and post-mortem [27], [28], it is apparent that certain structures show greater variability across different scanning and processing conditions and need to be analyzed with great caution. We can hypothesize that the differences observed between the two field strengths are related to the reduced gray/white matter contrast in T1-weighted images in these regions. An increase in the field strength generally improves image contrast-to-noise ratio (CNR) and this leads to better accuracy of the WM surface and pial surface reconstruction. Heavily myelinated structures such as the primary sensory-motor and retro-splenial or primary visual cortices [29] show larger measurement variability across field strengths in our study and in the afore-mentioned publications. Previous literature [30] has shown that myelin content is inversely correlated with intracortical circuit complexity. Because of this, highly myelinated cortex tends to be thinner (less than 2 mm in some areas as shown here, with the exception of the primary sensory-motor cortex) and the standard 1 mm isotropic resolution might be too coarse to properly segment the cortical ribbon. In order to overcome such errors and improve the accuracy of cortical surface reconstructions, the Human Connectome Project suggests acquiring 0.7 mm isotropic T1 and T2 weighted images [31].

### Statistical methodology

We used multivariate regression models to assess the relationship between cortical thickness and field strength. This method treats all regional cortical thickness as a vector of dependent variables and compares the mean vectors between the two field strengths (1.5T vs. 3.0T). This method has an advantage of controlling the overall probability of Type I error for these comparisons when there are a number of multiple comparisons. For example, if the Pillai's trace test does not find a statistically significant difference between the two mean vectors, then there is no need for comparison of each component of the vector of cortical thickness. However, if the Pillai's trace test is found to be statistically significant, then one should go ahead and identify components of the vector of cortical thickness that have resulted in a significant mean difference between the two vectors. The Pillai's trace test assumes that the data is normal and that the variance is the same in each group. Out of the 68 regions that we examined in this study, 16 regions may not have completely met the assumption of normality (data not shown). All other regions followed normal distribution according to Kolmogorov-Smirnov test. In addition, according to the central limit theorem the sampling distribution of mean of the regional cortical thickness will be approximately normally distributed given the large sample size of 295.

### Conclusions

In summary, our results in this study indicate that the effect of field strength is a more significant contributor to the observed cortical thickness than gender, scanner type, image resolution or pulse sequence. With the steady increase in the use of 3 T MRI scanners and the increased number of multi-center trials to understand brain morphometry, there is a need to understand how the estimation of cortical thickness is affected by extrinsic factors (field strength, image quality) for a proper interpretation of cortical thickness on age and gender.

### Limitations

In an ideal setting, this large sample of subjects would have been scanned at both 1.5T and 3T to assess the field dependence of cortical thickness in a controlled experiment. But such data is not available to us. In spite of these limitations we believe that these are the first comprehensive studies that explored the effect of field strength, gender, and age on regional and global cortical thicknesses on a relatively large cohort of normal subjects.
